# Gain Enhancement and Cross-Polarization Suppression of Cavity-Backed Antennas Using a Flared Ground Cavity and Iris

**DOI:** 10.3390/s23094389

**Published:** 2023-04-29

**Authors:** Yanxia Liu, Dustin Isleifson, Lotfollah Shafai

**Affiliations:** 1 Department of Electrical & Computer Engineering, University of Manitoba, Winnipeg, MB R3T 5V6, Canada; 2 Centre for Earth Observation Science, University of Manitoba, Winnipeg, MB R3T 2N2, Canada

**Keywords:** flare, iris, cavity-backed antenna, dual-polarized, differentially-fed, wideband, cross-polarization suppression, gain enhancement

## Abstract

Herein, we present new design principles for gain enhancement and cross-polarization suppression in dual-polarized cavity-backed antennas and demonstrate the capability in an octagonal cavity-backed open prism antenna (OCROP). In our approach, the gain is enhanced through an optimal flaring procedure and a novel metallic iris is used to control the electromagnetic fields and thereby reduce the cross-polarization. Previously, we investigated a dual-polarized OCROP antenna configuration and were able to simultaneously achieve 50% impedance bandwidth, 40% cross-polarization bandwidth (≤25 dB), and 10.2 dBi peak gain. In this study, we investigated gain enhancement by flaring an upper section of the ground cavity sidewalls, while maintaining a constant cavity height. Two cases were investigated: (1) the flare angle was modified, while the ratio of the non-flared to flared sidewall heights was kept constant, and (2) the ratio of the non-flared to flared sidewall heights was varied. In case 1, we established that, while increasing the flare angle results in a gain increase, there is a limit, as cross-polarization at the upper operating frequencies increases. In case 2, we were able to reduce the aperture phase error and achieve a higher peak gain of 12.8 dBi. To address the increased cross-polarization at the high frequency end when a large flare was used, we added a metallic iris at the junction of non-flared and flared sidewalls. We showed that increasing the iris width generally decreases the cross-polarization at high frequencies, without compromising the gain and impedance bandwidth. At an optimal width, it provides a nearly constant, low cross-polarization (below −25.8 dB) and a peak gain of 13.3 dBi, across the entire 50.7% impedance bandwidth of the antenna. We fabricated and successfully tested a prototype to verify the design and simulation approach. These results prove that incorporating an aperture flare with a metallic iris can significantly improve the gain and cross-polarization performance of cavity-backed antennas.

## 1. Introduction

Cavity-backed antennas are used in applications where compact structures are needed and stable high gain within a wide operating frequency band is desired [1] In recent years, such miniaturized antennas have been used on drones and in other remote sensing applications. They need to be compact, have low mass, and be dual-polarized, while having a wide impedance bandwidth, low cross-polarization, and high antenna gain. Cavity-backed antennas are good candidates, as they can readily be designed and adapted to meet these applications. In [2], we presented a dual-polarized L-band octagonal cavity-backed radiating open prism antenna (we called it OCROP antenna) with the above-mentioned desirable features. It was compact, lightweight, and had excellent frequency scalability [3] and a wide impedance bandwidth of >50%. We also achieved low cross-polarization (peak ≤ −25 dB, i.e., a low cross-polarization standard for many remote sensing applications) and moderately high peak gain of 10.2 dBi. In this paper, we aim to make the OCROP antenna more attractive for the applications of interest by further increasing its gain and suppressing its peak cross-polarization to ≤−25 dB at all operating frequencies, without compromising other important performance parameters such as the impedance bandwidth. 

There are a few gain enhancement techniques in the literature that can be applied to the OCROP antenna design, e.g., forming an antenna array [4,5,6,7] adding partially reflective surfaces (PRSs) above the aperture opening [8,9], and adding a flare to the original aperture opening [1,10]. Although the first two techniques are effective in improving the gain, they have some drawbacks. For instance, the array configuration requires a complex feed network, and the conventional PRS loading has an inherent disadvantage of narrow gain bandwidth [8,9]. In addition, PRS loading does not provide cross-polarization suppression. Although an array with image feed configuration offers cross-polarization suppression [6], to design an image feed for an array that requires differential and orthogonal feeding for each of its elements is very challenging as it often requires multi-layer topologies. The third technique, i.e., flaring, has been widely used for gain improvement of aperture type of antennas. For instance, the gain of a rectangular open-ended waveguide can be increased by adding a flare to its open end and forming a horn antenna [10], and in [1], a cavity-backed antenna employed a composite circular ground cavity with a flared lower section and a non-flared upper section to attain high gain for a wide impedance bandwidth. The maximum gain of an antenna increases with its aperture size compared to wavelength [11], provided that the aperture phase distribution remains uniform [4,12]. Adding a flare to the original aperture opening enlarges the aperture size, and in the meantime, induces co- and cross-polarized currents on the flare. It also increases the electrical path lengths away from the antenna axis and introduces aperture phase error that counters the gain enhancement. Thus, this method of gain enhancement is simple, but limited to small flare angles. The impact of flaring on the impedance bandwidth is usually minimal [1]. Therefore, a flared configuration was chosen due to its capacity for providing simultaneous support for high gain and wideband operation.

Although introducing a flare can potentially improve the gain, over-flaring can cause an imbalance of electric and magnetic field at the aperture that can increase the cross-polarization, especially at high frequencies [13]. Since this work has a high metric for cross-polarization (i.e., peak cross-polarization ≤ −25 dB over the entire $S_{\mathrm{dd}11}$ ≤ −10 dB impedance bandwidth within −90° ≤ θ ≤ 90° and 0° ≤ φ ≤ 360°), we have to remedy the potential consequence of increased cross-polarization. In this work, we propose a human eye inspired metallic iris to be added to the flared configuration to overcome this problem. The human eye iris regulates the amount of light that enters the eye by making the pupil larger (dilated) or smaller (constricted) [14] Similarly, a metallic iris, at the base of the flared section, can control the electromagnetic waves passing through it, and the balance of the electric and magnetic fields at the aperture [15,16,17,18]. With this added degree of freedom for regulating the co- and cross-polarized radiation, we can potentially reduce cross-polarization.

This paper incorporates two design concepts, i.e., adding a flare and adding an iris, to our original OCROP design to further improve its gain and cross-polarization performance without compromising its impedance bandwidth. It is worth mentioning that the cavity-backed configuration is applicable to many other antennas beyond the metallic open prism presented here. Therefore, the enhancement techniques investigated in this work have the potential to be applied to a wide range of antennas, e.g., dielectric resonator antennas [19,20,21], patch antennas [6], and horns [22]. Section 2 and Section 3 demonstrate several numerical studies on critical parameters in the two designs, which achieve optimal antenna performance, i.e., maximum gain enhancement with largest impedance/cross-polarization overlapping bandwidths. To verify the simulation results, we fabricated a flared antenna prototype, and compare the simulation and measurement results in Section 4. Lastly, we present our conclusions in Section 5.

## 2. OCROP-Flared Antenna

### 2.1. Antenna Configuration

**Figure 1:**
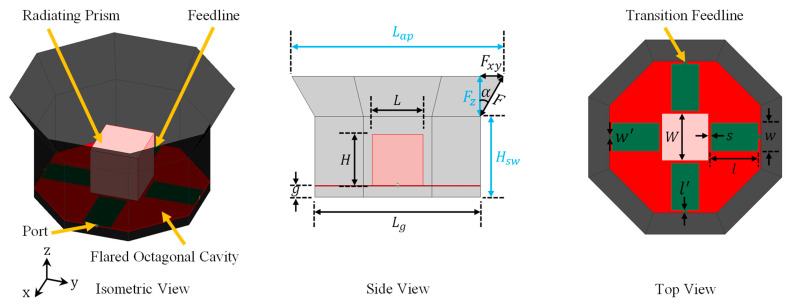
Geometry of the flared octagonal cavity-backed radiating open prism (OCROP-Flared) antenna.

Our goal is to further improve both the gain and the cross-polarization performance of our previous OCROP antenna from [2] without compromising its impedance bandwidth. As aperture flaring has the capacity for gain enhancement with minimal impact on the impedance bandwidth, it was adopted to meet the above-mentioned design goal. The OCROP design depended on resonance merging, in conjunction with coupling between the radiating feedlines and open prism to obtain a wide impedance bandwidth. The antenna excitation ports are located on the sidewalls of the ground cavity, and therefore, the feedline length (i.e., l + $l^{\prime }$) is a function of the aperture length. As a result, the impedance bandwidth varies with changes in the aperture length [2]. To avoid affecting the impedance matching, instead of flaring the entire ground cavity sidewalls, we add a flare to the aperture opening of the original non-flared ground cavity. In other words, only the upper section of the ground cavity sidewalls is flared, and the lower section remains non-flared. We call this new approach the “OCROP-Flared” design. Its geometry is shown in Figure 1. As can be seen, the flared sidewalls, with a length of *F*, tilt away from the *Z*-axis by a flare angle, α, on all eight sides. Same as the OCROP design, the open prism, sitting in the center of the ground cavity, is excited with two orthogonal pairs of differential feedlines to provide dual-polarization and low cross-polarization. The antenna consists of four parts: a conductive octagonal-shaped ground cavity, a conductive radiating cubic prism with no bottom face, two orthogonal pairs of differential feedlines, and a supporting dielectric substrate [2]. The only difference between an OCROP design and an OCROP-Flared design is the existence of the flared section of the ground cavity.

### 2.2. Numerical Analysis

In this section, we provide details on how flaring can be used to achieve gain and cross-polarization performance enhancement while maintaining wideband operation. The simulations in this paper were carried out in ANSYS Electronics Desktop 2021 R1©. Due to the perfect geometrical and electrical symmetry, the performance for the two orthogonal polarizations of interest (i.e., X- and Y-polarizations) are identical [2]. Therefore, in the following simulation studies, we only demonstrate the antenna performance under the Y-polarized operation mode by exciting the Y-polarized differential pair with equal amplitude and 180° out-of-phase signals while matching the X-polarized differential pair with 50 Ω loads.

#### 2.2.1. Effect of the Aperture Length

For the OCROP-Flared design, the two key parameters of the ground cavity, i.e., aperture length and cavity height, can be expressed as L_ap_ = L_g_ + 2F_xy_ and H_cavity_ = H_sw_ + F_z_. F_xy_ and F_z_ are the projections of the additional flare in the horizontal and vertical planes (see Figure 1), respectively. For flared sidewalls with a flare length *F* and flare angle α, the two projections are given by F_z_ = F∙cosα and F_xy_ = F∙sinα. To investigate the effect of the aperture length, we only vary the horizontal projection (*F_xy_*) and keep the vertical projection (*F_z_*) constant. Here, both the non-flared and the flared sidewall heights remained constant at H_sw_ = 80 mm and F_z_ = 40 mm, respectively. This leads to a cavity height of 120 mm (i.e., H_cavity_ = H_sw_ + F_z_ = 80 mm + 40 mm = 120 mm). As demonstrated in [2], this cavity height allows the lowest cross-polarization at the upper end of the operating frequency band. As will be elaborated below, this choice is critical for meeting the cross-polarization requirement in the OCROP-Flared design. Parametric studies on the aperture length were performed by increasing *L_ap_* from 165 mm to 285 mm in 20 mm steps. This corresponds to a gradual increase in flare angle from 0° up to 56.3°. At α = 0°, the antenna turns into an OCROP design. This case was included to demonstrate the superiority of an OCROP-Flared design with respect to an OCROP design of the same cavity height. All other antenna dimensions are kept constant as in [2], with L_g_ = 165 mm, L = W = H = 51 mm, g = 11 mm, s = 3 mm, l = 53 mm, w = 30 mm, l’ = 1 mm, and w’ = 3.6 mm.

**Figure 2:**
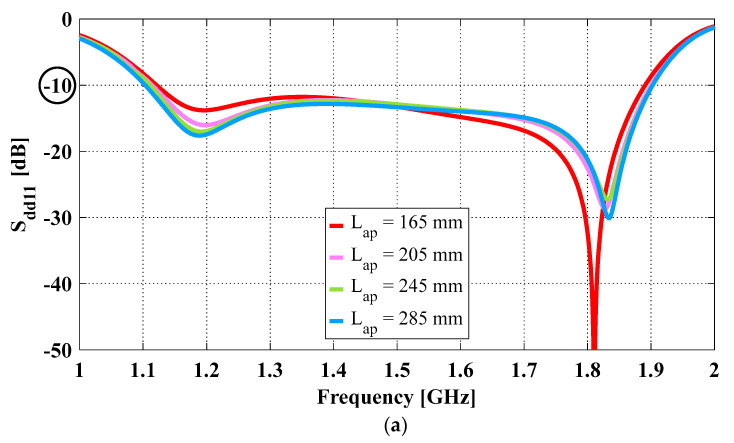

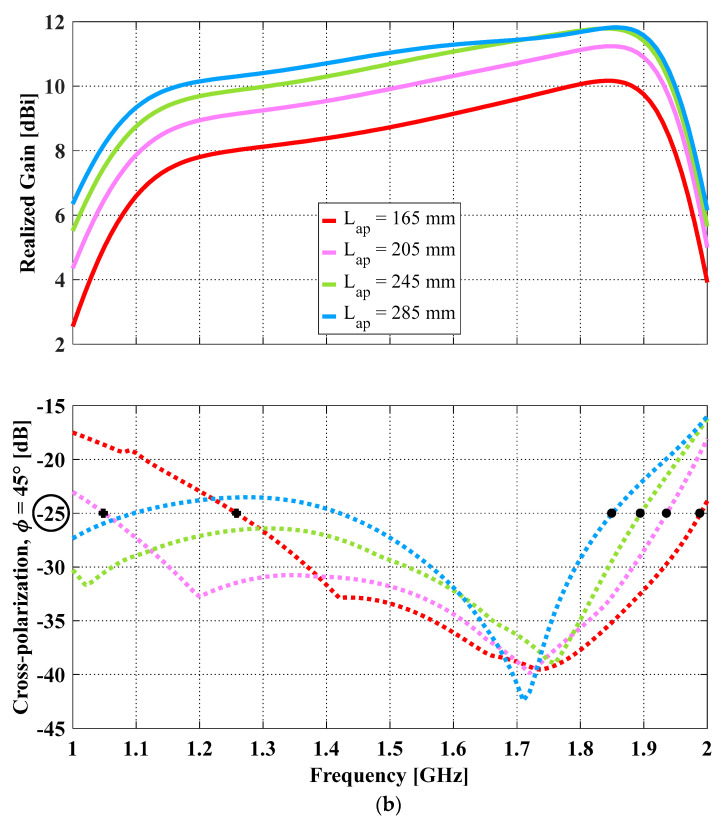
OCROP-Flared antennas with different aperture lengths (L_ap_): (**a**) Differential reflection coefficient (S_dd11_); (**b**) Realized gain (solid lines, top) and peak diagonal plane cross-polarization ratio (dotted lines, bottom; circular and cross markers represent the first and last frequencies that meet the peak cross-polarization ≤ −25 dB requirement, respectively).

Figure 2a shows the simulated differential reflection coefficient (i.e., S_dd11_) of the OCROP-Flared antennas with different aperture lengths. The fractional impedance bandwidths (i.e., imp. BW) of the antennas are calculated with the lower ($f_{1}$), upper ($f_{2}$), and center ($f_{c}$ = ($f_{1}$ + $f_{2}$)/2) frequencies for S_dd11_ ≤ −10 dB frequency bands (i.e., imp. BW = ($f_{2}$ − $f_{1}$)/$f_{c}$). As the aperture length increased from 165 mm to 285 mm, the impedance bandwidth showed a small increase, from 51.8% to 53.1%.

Simulated gain results are presented in Figure 2b (solid curves). As the aperture length increased from 165 mm to 285 mm, the average realized gain across the impedance bandwidth increased from 8.8 dBi to 10.9 dBi; the realized gain at 1.5 GHz (center frequency) increased from 8.7 dBi to 11.0 dBi (a 2.3 dB increase); the peak realized gain, which occurs around 1.85 GHz, increased from 10.2 dBi to 11.8 dBi (a 1.6 dB increase). As can be seen, increasing the aperture length resulted in a greater increase of gain at the lower end of the impedance bandwidth, while the gain increases at the upper end were more modest. As the aperture length increased to ≥ 265 mm, small gain reductions were observed at some of the upper-end frequencies. These gain reductions at upper frequencies are the results of increased aperture phase errors due to flaring [12].

The effect of varying the aperture length on the diagonal plane cross-polarization ratio is shown as the dotted curves in Figure 2b. Since the peak cross-polarization occurs in diagonal planes (i.e., φ = 45°, 135°) [2], we used the diagonal plane cross-polarization ratio as the metric for the evaluation of the cross-polarization performance. Same as [2], the frequency range for diagonal plane cross-polarization ≤ −25 dB is defined as the cross-polarization bandwidth (i.e., cross-pol BW). The overlap between the impedance and cross-polarization bandwidths is referred to as the combined bandwidth. Our goal is to maximize the combined bandwidth such that the cross-polarization requirement is met within the entire impedance bandwidth. To do this, we need to make sure that the cross-polarization bandwidth extends over to fully cover the impedance bandwidth.

The first and the last frequencies that meet the design criteria of peak cross-polarization ≤ −25 dB are referred to as $f_{\mathrm{first}}^{\mathrm{cross}-\mathrm{pol}}$ and $f_{\mathrm{last}}^{\mathrm{cross}-\mathrm{pol}}$ and marked as crosses and circles, respectively. As can be seen, both $f_{\mathrm{first}}^{\mathrm{cross}-\mathrm{pol}}$ and $f_{\mathrm{last}}^{\mathrm{cross}-\mathrm{pol}}$ shift down as the aperture length increases. For instance, $f_{\mathrm{first}}^{\mathrm{cross}-\mathrm{pol}}$ first drops below the low end of the operating frequency band (i.e., ~1.1 GHz) when L_ap_ increased to 205 mm, and then below the L-band (i.e., below 1 GHz) when L_ap_ increased to 225 mm. For the aperture length from 165 mm to 245 mm, the cross-polarization requirement is met within the entire range of f ∈ [$f_{\mathrm{first}}^{\mathrm{cross}-\mathrm{pol}}$, $f_{\mathrm{last}}^{\mathrm{cross}-\mathrm{pol}}$]. Therefore, the cross-polarization bandwidth for these cases is determined by $f_{\mathrm{first}}^{\mathrm{cross}-\mathrm{pol}}$ and $f_{\mathrm{last}}^{\mathrm{cross}-\mathrm{pol}}$. As the aperture length increased from 165 mm to 245 mm, the −25 dB cross-polarization bandwidth significantly increases from 44.9% to a maximum of 71.9%. This increase is due to $f_{\mathrm{first}}^{\mathrm{cross}-\mathrm{pol}}$ shifting down. For the two cases, namely L_ap_ = 205 mm and 225 mm (α = 26.6° and 36.9°, respectively), since $f_{\mathrm{first}}^{\mathrm{cross}-\mathrm{pol}}$ ≤ $f_{1}\;$ and $f_{\mathrm{last}}^{\mathrm{cross}-\mathrm{pol}}$ ≥ $f_{2}$, their cross-polarization bandwidth fully covers the corresponding impedance bandwidth, resulting in maximum combined bandwidths of 52.1% and 52.4%, respectively. As the aperture length further increases to 265 mm and 285 mm, the cross-polarization requirement is no longer met at some frequencies within the range of f ∈ [$f_{\mathrm{first}}^{\mathrm{cross}-\mathrm{pol}}$, $f_{\mathrm{last}}^{\mathrm{cross}-\mathrm{pol}}$]. For instance, when L_ap_ = 285 mm, the diagonal plane cross-polarization ratio increases to >−25 dB from 1.10 GHz to 1.41 GHz. This leads to significant decreases in cross-polarization bandwidth, and consequently, combined bandwidth (see Table 1). For instance, the cross-polarization and combined bandwidth reduced to 36.0% and 26.3% when the aperture length increased to 265 mm and 285 mm, respectively.

A summary of the performance comparison is given in Table 1. Regardless of the change in the flare angle, the impedance bandwidth remains wide. Gain improvement was observed across the entire operating frequency band as the aperture became larger. For the cases where L_ap_ = 205 mm and 225 mm, the −25 dB cross-polarization requirement is met across the entire impedance bandwidths.

To evaluate the overall improvement from flaring, an OCROP antenna (i.e., non-flared, L_ap_ = L_g_ = 165 mm) is compared with an OCROP-Flared antenna that has the largest combined bandwidth from Table 1 (where L_ap_ = 225 mm). The overall height of these two antenna configurations is the same, i.e., H_cavity_ = 120 mm. By flaring the upper section of the ground cavity sidewalls, we have increased the combined bandwidth of operation (i.e., operating frequency range where both S_dd11_ ≤ −10 dB and peak cross-polarization ≤ −25 dB are met) from 40.0% to 52.4%, and we have also increased the average gain across the combined bandwidth from 9.1 dBi to 10.3 dBi. This is a significant performance improvement. Further gain enhancement can be obtained, at the cost of reduced overlapping bandwidth. For example, with L_ap_ = 265 mm, the combined bandwidth is 36.0% due to cross-polarization being slightly higher than −25 dB from 1.26 to 1.31 GHz, while the average gain across the combined bandwidth is 11.1 dBi (an increase of 2.0 dB compared to the non-flared case with the same cavity height).

#### 2.2.2. Effect of the Ratio of the Non-Flared and Flared Cavity Sidewall Heights 

The above study on the aperture length was performed under the condition that the cavity height was fixed at $H_{\mathrm{cavity}}$ = $H_{\mathrm{sw}}$ + $F_{z}$ = 120 mm by keeping both non-flared and flared sidewall heights constant at $H_{\mathrm{sw}}$ = 80 mm and $F_{z}$ = 40 mm, respectively. However, the same cavity height can also be realized with different combinations of the non-flared and the flared sidewall heights. For example, all six pairs of non-flared and flared sidewall heights shown in Table 2 result in the same cavity height of 120 mm. To investigate the effect of varying the ratio of the two heights, i.e., $H_{\mathrm{sw}}/F_{z}$, the aperture length and the overall cavity height are kept constant at $L_{\mathrm{ap}}$ = 265 mm and $H_{\mathrm{cavity}}$ = 120 mm, respectively. 

**Figure 3:**
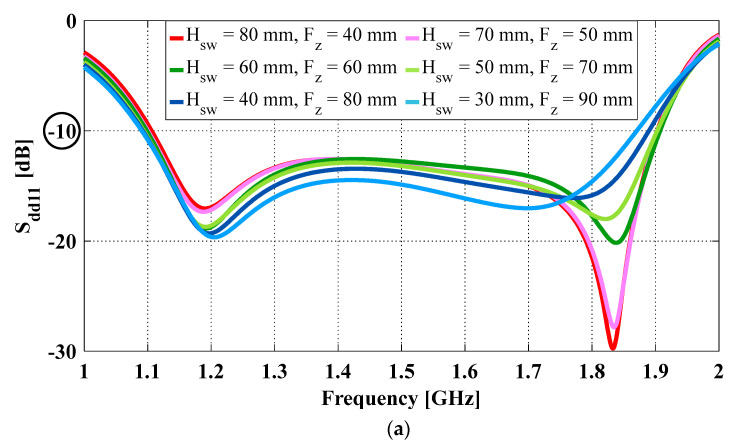

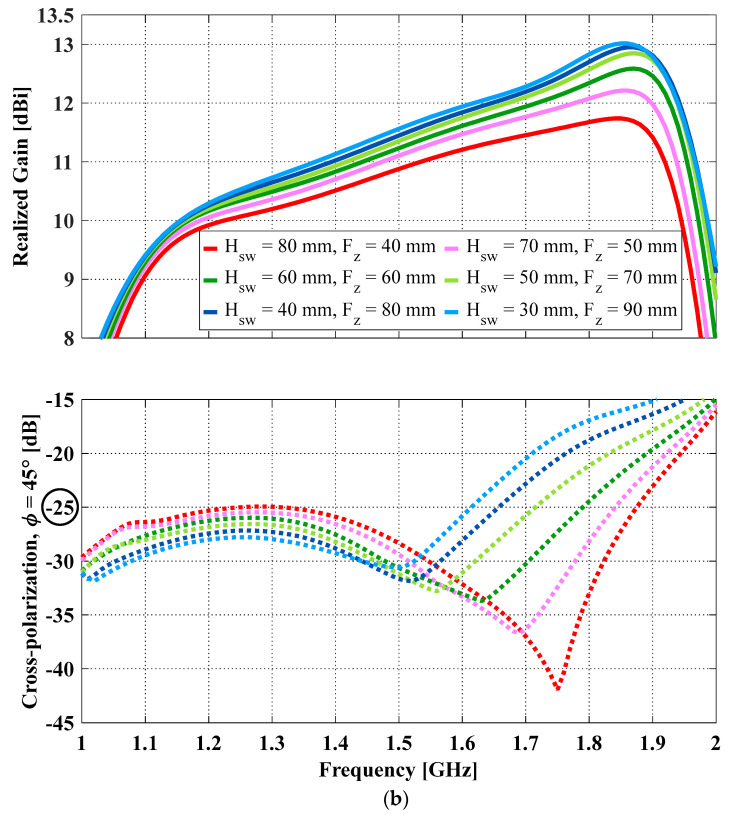
OCROP-Flared antennas with different combinations of non-flared and flared ground cavity sidewall heights when $L_{\mathrm{ap}}$ = 265 mm and $H_{\mathrm{cavity}}$ = 120 mm: (**a**) Differential reflection coefficient (S_dd11_); (**b**) Realized gain (solid lines, top) and peak diagonal plane cross-polarization ratio (dotted lines, bottom).

The performance comparison of the antennas with the six pairs of $H_{\mathrm{sw}}$ and $F_{z}$ is shown in Figure 3 and summarized in Table 2. It shows that, when the overall cavity height and aperture length are fixed, varying the ratio of the non-flared to the flared sidewall heights has little impact on the impedance bandwidth, but its effects on both realized gain and peak diagonal plane cross-polarization are noticeable. As the non-flared sidewalls become shorter and the flared ones become taller, the flare angle α becomes smaller, and the aperture phase error decreases. As a result, the rate of increase in the antenna gain increases, especially at the upper end of the impedance bandwidth. For instance, the peak realized gain, which occurs around 1.85 GHz, is 11.7 dBi when $H_{\mathrm{sw}}$ = 80 mm and $F_{z}$ = 40 mm (i.e., α = 51.3° and F = 64.0 mm). It goes up to 13.0 dBi when $H_{\mathrm{sw}}$ = 50 mm and $F_{z}$ = 70 mm (i.e., α = 35.5° and F = 86.0 mm). This is a 1.3 dB increase in peak gain with the same aperture size and cavity height.

**Figure 4:**
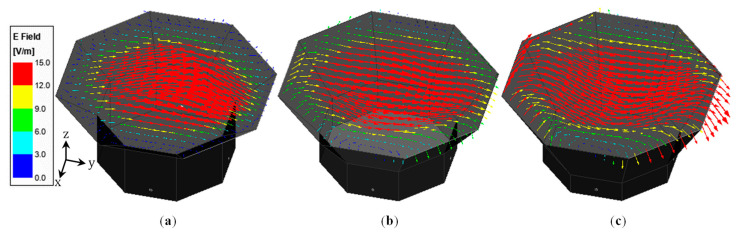
E-field distribution for 1.87 GHz at the aperture opening of OCROP-Flared antennas with different flare heights ($L_{\mathrm{ap}}$ = 265 mm and $H_{\mathrm{cavity}}$ = 120 mm): (**a**) $H_{\mathrm{sw}}$ = 80 mm and $F_{z}$ = 40 mm; (**b**) $H_{\mathrm{sw}}$ = 50 mm and $F_{z}$ = 70 mm; (**c**) $H_{\mathrm{sw}}$ = 30 mm and $F_{z}$ = 90 mm. (The open prism, feedlines and feedlines are not shown. All three cases share the same color key and oblique view.).

As for the peak diagonal plane cross-polarization ratio, reducing the $H_{\mathrm{sw}}$ to $F_{z}$ ratio improves it at the lower end of the impedance bandwidth and significantly degrades it at the upper end. For example, when $H_{\mathrm{sw}}$ = 80 mm and $F_{z}$ = 40 mm, the cross-polarization ratio at 1.27 GHz (i.e., at the lower end of the impedance bandwidth) is −24.9 dB and at 1.87 GHz (i.e., at the upper end of the impedance bandwidth) is −25.4 dB; when $H_{\mathrm{sw}}$ = 50 mm and $F_{z}$ = 70 mm, the corresponding cross-polarization at the lower end reduces to −26.6 dB (i.e., <−25 dB) and the one at the upper end increases to −18.8 dB (i.e., >−25 dB). To understand the reason for increased cross-polarization at upper frequencies, E-field distribution at the aperture opening of three antennas with different combinations of non-flared and flared sidewall heights, namely $H_{\mathrm{sw}}$ = 80 mm and $F_{z}$ = 40 mm, $H_{\mathrm{sw}}$ = 50 mm and $F_{z}$ = 70 mm, and $H_{\mathrm{sw}}$ = 30 mm and $F_{z}$ = 90 mm, was plotted for 1.87 GHz and compared in Figure 4. The aperture length and cavity height for these three cases were kept constant at $L_{\mathrm{ap}}$ = 265 mm and $H_{\mathrm{cavity}}$ = 120 mm. It shows that, as the flare height increases, the radiation from the edges of the aperture opening becomes more pronounced. These fields at the edges consist of both co- and cross-polarized components, and they are non-uniform and asymmetric. As a result, the cross-polarized components cannot be cancelled out, leading to higher cross-polarization at the sampled frequency.

The phenomenon of increased cross-polarization at the upper frequencies was also observed in Section 2.2.1. In that study, it occurred as the aperture size became larger, which corresponds to a larger flare angle and a longer flare length. In this study, it occurred when the $H_{\mathrm{sw}}$/$F_{z}$ ratio became smaller, which corresponds to a smaller flare angle and a longer flare length. By combining these two observations, it is reasonable to conclude that the cross-polarization increase at the upper frequencies is a result of increased flare length. To take the advantage of the gain enhancement due to a smaller $H_{\mathrm{sw}}$/$F_{z}$ ratio (i.e., a smaller flare angle and a longer flare length), we need to overcome the resulting challenge of high cross-polarization at the upper end of the impedance bandwidth, without compromising the already achieved gain improvement. In the following section, we present a novel and elegant solution that draws upon inspiration from the human eye—an iris structure.

## 3. OCROP-Flared-Iris Antenna

**Figure 5:**
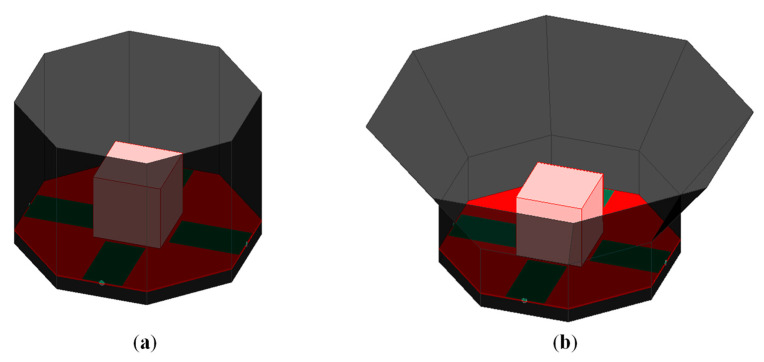

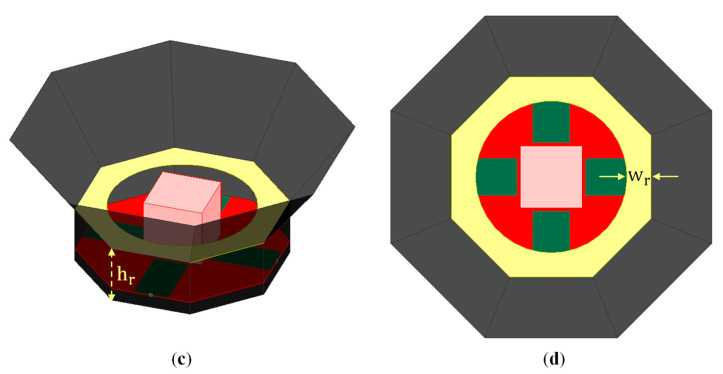
Configurations of three differentially fed dual-polarized octagonal cavity-backed radiating open prism antennas with the same cavity height of $H_{\mathrm{cavity}}$ = 120 mm: (**a**) Antenna #1 (without flare and iris, i.e., OCROP); (**b**) Antenna #2 (with flare and without iris, i.e., OCROP-Flared); (**c**) Trimetric and (**d**) top views of Antenna #3 (with flare and iris (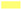), i.e., OCROP-Flared-Iris). (The iris is shorted to the inner junction of the non-flared and flared ground cavity sidewalls).

To remedy the problem of the increased cross-polarization in an OCROP-Flared antenna with a small $H_{\mathrm{sw}}$/$F_{z}$ ratio, this section presents and investigates a new “iris” design concept where a metallic iris is added to the inner junction of the non-flared and flared ground cavity sidewalls, as shown in Figure 5c,d. We have named this design a “flared octagonal cavity-backed radiating open prism with an iris (OCROP-Flared-Iris)”, or Antenna #3. To demonstrate its superiority, we compare it with our two previous designs, i.e., OCROP (i.e., Antenna #1, without flare and iris, shown in Figure 5a) and OCROP-Flared (i.e., Antenna #2, with flare and without iris, shown in Figure 5b).

All three antennas have the same cavity height of 120 mm: Antenna #1 only has non-flared sidewalls, i.e., $H_{\mathrm{cavity}}$ = $H_{\mathrm{sw}}$ = 120 mm (i.e., α = 0° and F = 0 mm), while Antennas #2 and #3 have both non-flared and flared sidewalls, i.e., $H_{\mathrm{cavity}}$ = $H_{\mathrm{sw}}$ + $F_{z}$ = 50 mm + 70 mm = 120 mm (i.e., α = 35.5° and F = 86.0 mm). Antenna #1 has a smaller aperture length of 165 mm, i.e., L_ap_ = L_g_ = 165 mm, and Antennas #2 and #3 have a larger aperture length of 265 mm, i.e., L_ap_ = L_g_ + 2F_xy_ = 165 mm + 2*50 mm = 265 mm, due to the flaring of the upper section of the ground cavity. These dimensions are summarized in Table 3. All other antenna dimensions are kept constant.

To provide maximum cross-polarization reduction, the iris is octagonal on the outside, which ensures that it is perfectly shorted to the ground cavity sidewalls, and circular on the inside to avoid sharp corners that induce cross-polarization [2]. Since the cross-polarization at the higher frequencies is mostly due to the abrupt junctions of the ground cavity, the placement of the iris should be able to block the waves that were reflected off of these locations. To determine the optimum location for the iris, extensive simulation studies have been performed. The simulation results show that the optimum iris location for maximum cross-polarization reduction is at the inner junction of the non-flared and flared sidewalls, i.e., $h_{r}$ = $H_{\mathrm{sw}}$ (as shown in Figure 5c). Since the shape and the location of the iris are fixed, the only other critical parameter is its width, $w_{r}$. Due to the combination of an octagonal outer edge and a circular inner edge, the iris is the narrowest at the center of each octagon side and widest at the octagon corners. In the following discussion, the iris width refers to its narrowest width, namely $w_{r}$ (as shown in Figure 5d). The iris of a human eye regulates the amount of light that enters the eye by making the pupil larger (dilated) or smaller (constricted) [14] Similarly, the metallic iris in Antenna #3 controls the amount of electromagnetic waves passing through it by varying its width ($w_{r}$). 

**Figure 6:**
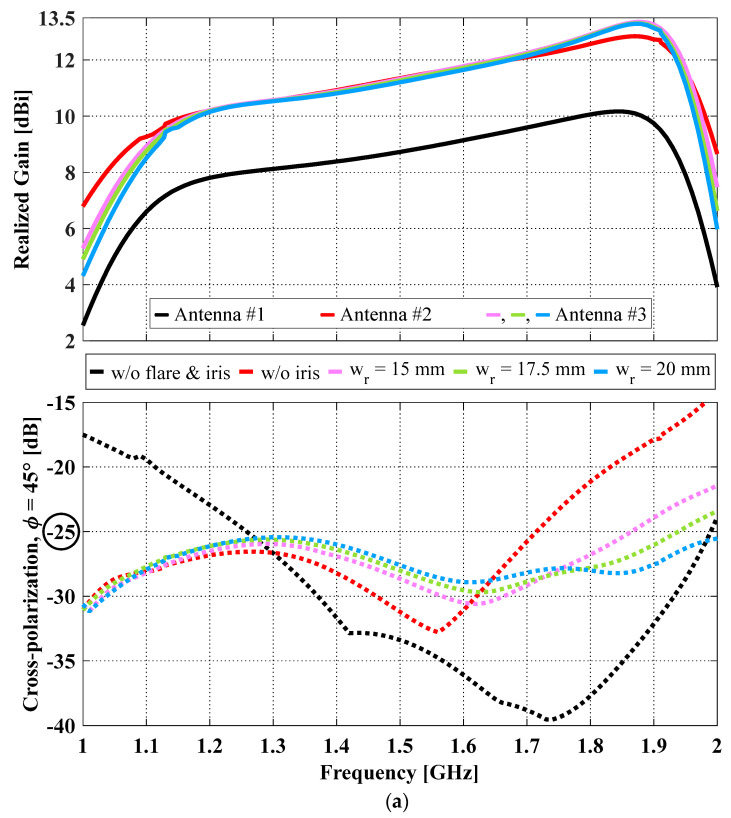

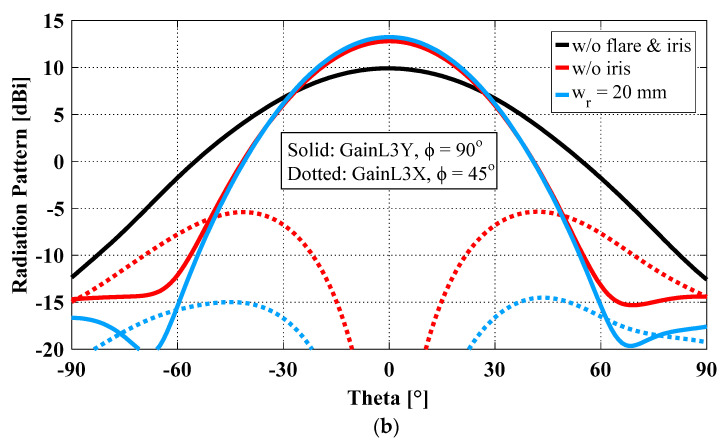
(**a**) Realized gain (solid) and diagonal plane cross-polarization (dotted) of OCROP antennas without flare and iris (Antenna #1, black), and with flare but without iris (Antenna #2, red), and with flare and iris of different ring widths, w_r_, (Antenna #3, yellow, green, blue), and (**b**) radiation patterns of the E-plane co-pol (RealizedGainL3Y, solid) and diagonal plane cross-polarization (RealizedGainL3X, dotted) of Antennas #1 (black), #2 (red) and #3 (w_r_ = 20 mm, blue) at 1.89 GHz.

To study the effect of the iris width, we varied it from 15 mm to 20 mm with a step of 2.5 mm while all other antenna dimensions were kept constant. The realized gain and the peak diagonal plane cross-polarization ratio of the antennas with (Antenna #3, $L_{\mathrm{ap}}$ = 265 mm and $H_{\mathrm{cavity}}$ = 120 mm) and without an iris (Antenna #2, $L_{\mathrm{ap}}$ = 265 mm and $H_{\mathrm{cavity}}$ = 120 mm) are compared in Figure 6a. To demonstrate the combined effects of the flaring and the iris, the results for the antenna without either flaring or iris (Antenna #1, $L_{\mathrm{ap}}$ = 165 mm and $H_{\mathrm{cavity}}$ = 120 mm) were also plotted for comparison. Different colors represent different designs, i.e., black for Antenna #1, red for Antenna #2, and yellow, green, and blue for Antenna #3 with three different iris widths. Solid and dotted curves represent realized gain and cross-polarization ratio, respectively. As expected, due to flaring, Antenna #2 has higher realized gain within the entire impedance bandwidth and lower cross-polarization in the lower end of the impedance bandwidth (below 1.3 GHz) than Antenna #1. However, its cross-polarization at frequencies above 1.3 GHz is higher than that of Antenna #1. For instance, at frequencies > 1.71 GHz, the cross-polarization of Antenna #2 has exceeded the design requirement of ≤−25 dB. The comparison between Antennas #2 and #3 shows that the addition of an iris is very effective in reducing the cross-polarization at the upper end of the impedance bandwidth (≥1.64 GHz). For example, without an iris, the cross-polarization ratio at 1.89 GHz is −18.2 dB; by adding an iris of $w_{r}$ = 20 mm, the cross-polarization ratio at the same frequency was reduced to −27.7 dB (i.e., a 9.5 dB reduction). 

In the cases of $w_{r}$ = 17.5 and 20 mm, the lower limit of their cross-polarization bandwidth ($f_{\mathrm{first}}^{\mathrm{cross}-\mathrm{pol}}$) remained well below the lower limit of their impedance bandwidth ($f_{1}$), and the upper limit of their cross-polarization bandwidth ($f_{\mathrm{last}}^{\mathrm{cross}-\mathrm{pol}}$) shifted above the upper limit of their impedance bandwidth ($f_{2}$). As a result, in these two cases, we have achieved the largest impedance/cross-polarization overlapping bandwidths (i.e., combined BW = imp. BW, or the cross-polarization requirement of ≤−25 dB was met within the entire impedance bandwidth). Although more cross-polarization reduction can be achieved at the upper-end frequencies by further increasing the iris width, increasing it beyond 20 mm will slightly raise the cross-polarization at the lower-end frequencies around 1.3 GHz above −25 dB, resulting in a drastic decrease in the cross-polarization bandwidth and consequently a much smaller combined bandwidth. 

We note that the addition of an iris with the three sampled iris widths does not lead to a compromise in gain, as compared in Table 4. On the contrary, when an iris of any of the three sampled iris widths was added to Antenna #2, 0.2 dB (from 11.3 dBi to 11.5 dBi) and 0.5 dB (12.8 dBi to 13.3 dBi) increases in the average and peak realized gains across the impedance bandwidth, respectively, were observed. The increased gain is due to the iris transferring the cross-polarized radiation to co-polarized radiation. The radiation patterns for the E-plane co-pol (i.e., GainL3Y at φ = 90°) and the diagonal plane cross-polarization (i.e., GainL3X at φ = 45°) of the three antennas at 1.89 GHz are shown in Figure 6b. At 1.89 GHz, the E- and H-plane half power beamwidths (HPBWs) of the antenna without flare and iris are 59° and 60°, respectively; with the flaring alone or a combination of flare and iris, the two corresponding HPBWs decrease to 39° and 43°, respectively. As expected, gain enhancement leads to beamwidth reduction. Having a narrow beamwidth is critical for applications such as microwave remote sensing to achieve high resolution mapping [23]. As for the impedance bandwidth, the addition of an iris slightly reduces the impedance bandwidth, as shown in Table 4, and the bandwidth reduction increases as the iris width becomes wider. For instance, the impedance bandwidth of the flared antenna without an iris is 53.9%, and this number reduces to 51.5%, 50.7%, and 49.7% when $w_{r}$ = 15, 17.5 and 20 mm, respectively. Further increases in the iris width will result in the S_dd11_ slightly going above −10 dB in the mid-section of the operating frequency band, and consequently an even narrower impedance bandwidth.

**Figure 7:**
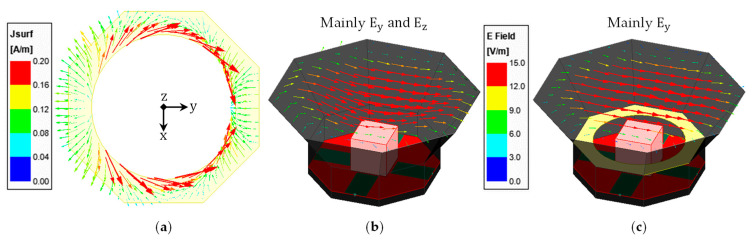
(**a**) Current distribution on the iris of w_r_ = 20 mm at 1.89 GHz, and E-field distribution at the aperture opening of the antenna (**b**) without and (**c**) with the iris of w_r_ = 20 mm at 1.89 GHz, respectively. ((**b**,**c**) share the same color key between them).

To demonstrate how the iris contributed to the improvement in the co- and cross-polarized radiation, the current distribution (Figure 7a) on the iris and the E-field distribution at the aperture opening of the antenna without (Figure 7b) and with (Figure 7c) an iris (w_r_ = 20 mm) were plotted for 1.89 GHz. As can be seen, the two major current components are along the *X* and *Y*-axes, and the current distribution is almost symmetric along the Y-axis (i.e., co-pol direction). This leads to the majority of the X-directed current components cancelling each other, and as a result, the cross-polarization is reduced. The E-field distribution at the aperture opening of the antenna without the iris clearly shows a strong presence of both co- and cross-polarized fields (namely, *E_y_* and *E_z_*), while the fields at the aperture opening of the one with the iris are mainly co-polarized (*E_y_*).

## 4. Antenna Prototype Fabrication and Test Results

**Figure 8:**
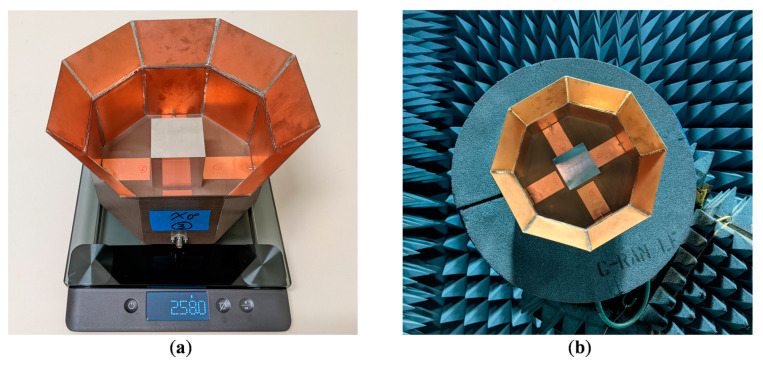
OCROP-Flared prototype: (**a**) Placed on mass scale (the scale reads 258.0 g); (**b**) In the compact antenna test range for radiation patterns.

To experimentally verify the validity of the simulation results, we fabricated and tested a prototype of the differentially-fed, dual-polarized OCROP-Flared antenna, as shown in Figure 8. The fabrication process is the same as that of the base design, i.e., OCROP, from [2]. For the construction of the ground cavity, instead a copper or aluminum plate, we used a one-sided printed circuit board (PCB) due to its light weight and excellent solderability. This gave us the freedom to choose any dielectric substrate to construct the ground cavity because the effects of material properties on the antenna performance were minimal. Based on the material availability at our fabrication facility, we used a 0.762 mm thick DiClad 527 ($\varepsilon_{r}$ = 2.5 and tanδ = 0.0018). The open prism of 51 mm × 51 mm × 51 mm is a hollow cube with no bottom face. It has a wall thickness of 0.4 mm and was milled from an aluminum cube. Same as in the simulation, we used a 0.762 mm thick one-sided Rogers CuClad 217 to construct the feedlines and support the open prism. The supporting substrate was suspended 11 mm above the ground plane (i.e., g = 11 mm). To maintain such a constant gap, a 11 mm thick octagonal shaped foam with the same diameter as the ground plane was placed in the gap. To allow easier alignment and assembly, a 51 mm × 51 mm square relief was routered half deep into the center of the supporting substrate where the open prism was placed. Four small stainless-steel screws were used in each bottom corner of the open prism to attach it to the supporting substrate. The vertical projection of the extension flare was F_z_ = 40 mm and the flare angle was set to α = 30°, which resulted in an aperture length of L_ap_ = 211.2 mm. Table 5 summarizes the detailed antenna dimensions (reference dimensions can be found in Figure 1).

**Figure 9:**
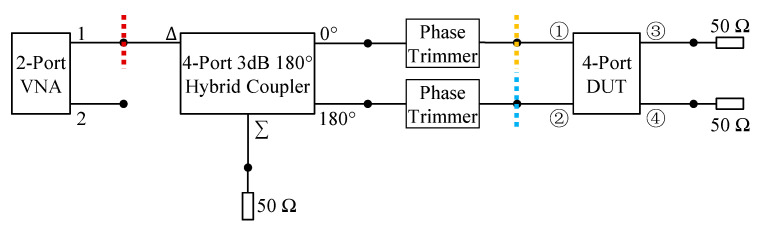
Feed network setup. (Ports ① and ② are excited for Y-polarization; ports ③ and ④ are excited for X-polarization.)

To provide differential feeding to the antenna, we used the same feed network from [2]. It consists of four parts: a 1–2 GHz 3 dB 180° hybrid coupler, phase trimmers, 50 Ω coaxial cables, and 90° angled SMA adaptors. The coupler is a 4-port device which divides the input signal from its differential port (△) into two output signals of equal amplitude and opposite phases at its port 0° and port 180°. The feed network setup is presented in Figure 9. As can be seen, the differential port of the coupler was connected to a Vector Network Analyzer (VNA), and its ports 0° and 180° were connected to phase trimmers, 50 Ω cables, and then to the antenna ports 0° and 180°. Phase trimmers were used such that the phase difference between the two input signals into antenna differential ports are close to the ideal value of 180°. With the phase trimmers, there was only a ≤ 5° differential phase error for the 1.0~1.9 GHz frequency range. The magnitude imbalance between the two differential branches of the feed network was ≤0.5 dB (i.e., close to the ideal value of 0 dB) for the 1.05~2.0 GHz frequency range.

**Figure 10:**
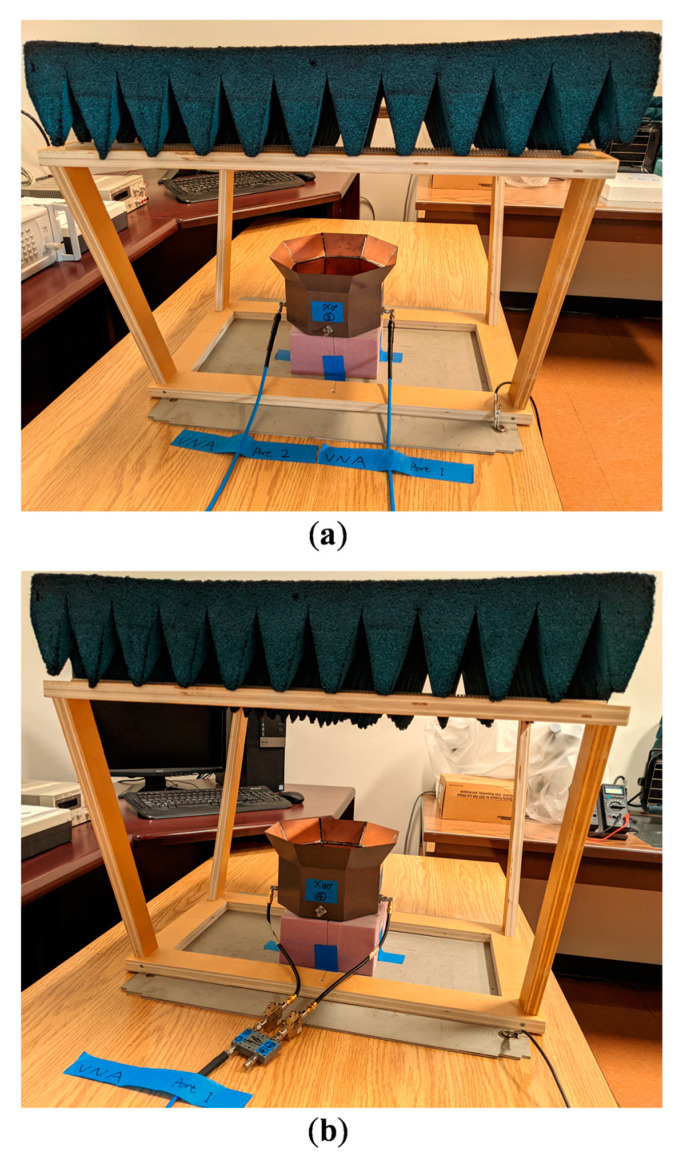
Two setups for the differential reflection coefficient measurements of the fabricated OCROP-Flared prototype: (**a**) without and (**b**) with the differential feed network.

**Figure 11:**
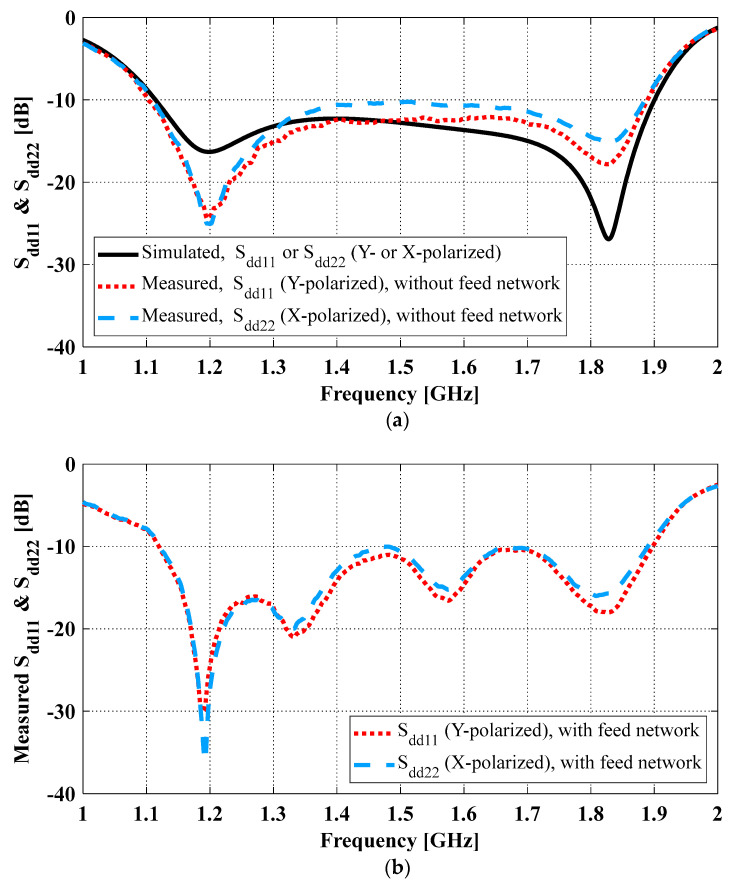
Simulated and measured differential reflection coefficients of the fabricated OCROP-Flared prototype: (**a**) Without and (**b**) with the differential feed network.

The differential reflection coefficients of the antenna were evaluated with a two-port VNA. Two types of measurements were conducted, and their measurement planes are indicated by the dashed lines in Figure 10. The first type (Figure 10a) obtained differential reflection coefficients from the post processing of the single-ended S-parameters measured at the antenna ports [24]. The measurement plane is indicated by the blue and yellow dashed lines in Figure 9. In this type of measurement, we assumed differential signals for one differential pair (1 V and −1 V) and matched loads for the other pair (0 V and 0 V). This is the same approach that HFSS used for computing the reflection coefficients. The only difference is that HFSS used the simulated single-ended S-parameters, instead of the measured ones. Both simulated and measured differential reflection coefficients using this method are shown in Figure 11a. The differential reflection coefficients for Y- and X-polarizations are S_dd11_ and S_dd22_, respectively. As can be seen, the simulated and measured impedance bandwidths for both S_dd11_ and S_dd22_ ≤ −10 dB agree well. The second type (Figure 10b) measured differential refection coefficients directly at the input of the feed network. The measurement plane is indicated by the red dashed line in Figure 9. The reflection coefficients measured with this approach are shown in Figure 11b. As can be seen, compared with the simulated result without the feed network (black solid curve in Figure 11a), there is good agreement in terms of the −10 dB impedance bandwidth. The discrepancy between the simulated and the measured results is mainly reflected in the number of the notches in the curves, which was attributed to the effects of the hybrid coupler. In all the cases (i.e., with or without the feed network, simulated or measured), a more than 50% impedance bandwidth was achieved. The two types of measured differential reflection coefficients, i.e., with and without the feed network, verified the effects of the feed network, similar to what was observed in [2]. 

**Figure 12:**
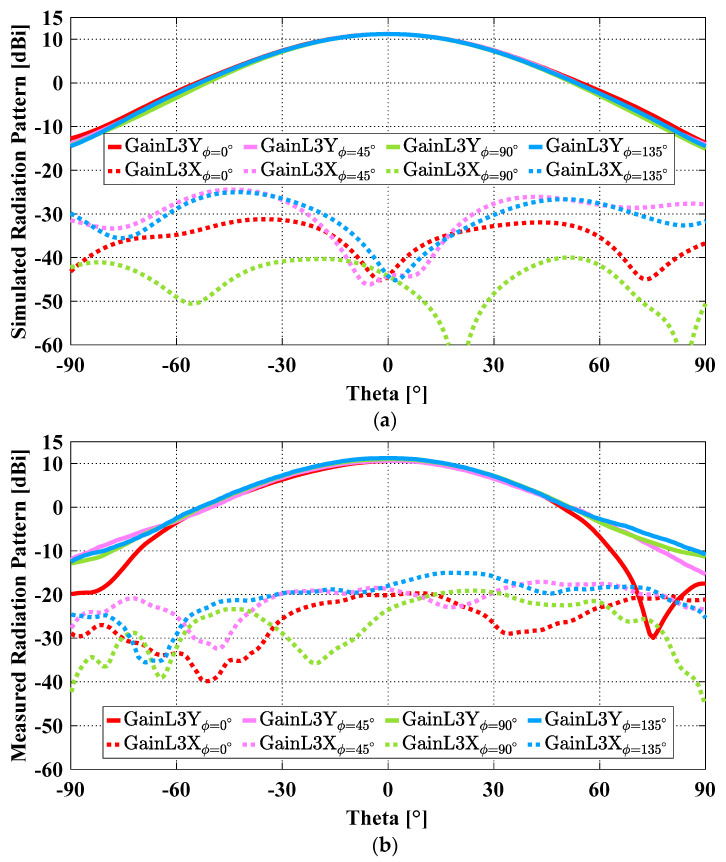
Simulated and measured radiation patterns of the OCROP-Flared protype at 1.8 GHz: (**a**) Simulated and (**b**) measured results.

Radiation patterns were measured in a Compact Antenna Test Range. Figure 12a,b compare the simulated and measured radiation patterns (accounting for the amplitude and phase errors) at 1.8 GHz in different φ-cut planes, namely principal planes (i.e., φ = 0°, 90°) and diagonal planes (i.e., φ = 45°, 135°). As can be seen, the simulated and measured gain are almost the same (11.2 dBi). However, there are some discrepancies between the simulated and measured cross-polarization results. In addition to the two contributors for cross-polarization errors discussed in [2] (i.e., the limit of the cross-polarization of the Compact Range reflector (−35 dB) and the scattering from the tower and the antenna mount on the tower), the asymmetry in the fabricated prototype could cause higher measured cross-polarization. It was difficult to maintain perfect symmetry of the flared region during the fabrication. However, the peak cross-polarization for the worst-case scenario (i.e., φ = 135°) is −26.3 dB which is still lower than the maximum acceptable cross-polarization level of −25 dB. Besides its attractive performance, the fabricated antenna also has some desirable physical attributes, such as being lightweight (258.0 g) and compact (1.056 λ × 1.056 λ × 0.6 λ at 1.5 GHz).

## 5. Conclusions

In this paper, we presented new design concepts for gain enhancement and cross-polarization suppression in dual-polarized cavity-backed antennas and demonstrated their capability in an octagonal cavity-backed open prism antenna (OCROP).

We investigated gain enhancement by flaring an upper section of the ground cavity sidewalls, while maintaining a constant cavity height (selected as 120 mm to demonstrate the concept). At the same time, we required that the gain improvements should not compromise the cross-polarization level or the impedance bandwidth. 

Two cases were investigated. In the first case, several aperture sizes for the flared section were examined, while keeping the height of the flared upper section constant at 40 mm. We showed that increasing the aperture size can increase the gain over the entire operating frequency band and reduce the cross-polarization at the lower frequencies. Since the height of the flared section was kept constant, a large aperture sizes required a large flare angle. This produced an aperture phase error and consequently, there was a limit in the gain performance improvement. To overcome this problem, in the second case, the height of the flared section was increased, while keeping the overall antenna height constant at 120 mm. This reduced the flare angle, and accordingly, the resulting aperture phase error was reduced. As a result, larger gains were obtained. In both cases, it was found that a longer flare length would cause cross-polarization deterioration at the upper frequencies.

To remedy the problem of increased cross-polarization, a novel idea of introducing a metallic iris at the base of the flared section, was investigated. Simulation studies showed that small iris widths decreased the cross-polarization at the upper frequencies. As much as 10 dB cross-polarization reduction was achieved. In particular, it was found that for an optimal iris width, the level of reduced cross-polarization could be maintained nearly constant, over the entire operating frequency band.

We fabricated and successfully tested a prototype to verify the design and simulation approach. These results prove that incorporating an aperture flare with a metallic iris can significantly improve the gain and cross-polarization performance of cavity-backed antennas.

The iris concept is a remarkable achievement in cross-polarization reduction that can be used in other aperture type antennas, such as horn antennas. With a combination of the flaring and the iris, the peak gain of the antenna was increased by 3.1 dB and its cross-polarization was suppressed to below −25.8 dB within the entire 50.7% impedance bandwidth. This is a significant performance enhancement, and we plan to investigate these approaches in future designs.

To demonstrate the advantages of the proposed antenna, we compared it with two wideband high gain antennas, i.e., a standard gain horn (operating from 1.12~1.7 GHz) from [22] and a patch array with image feed (operating from 9.4~10.7 GHz) from [6], in Table 6. As can be seen, compared to the standard gain horn that operates in a similar frequency range, our antenna is significantly smaller and lighter, and offers dual-polarization, wider impedance bandwidth, and higher aperture efficiency. In standard gain horns, they are not interested in diagonal plane cross-polarization where it usually peaks, so the information on peak cross-polarization is not supplied by manufactures. The patch array with image feed offers cross-polarization suppression beyond principle planes. However, it was designed to have low cross-polarization within half-power beamwidths, while our antenna was designed to have low cross-polarization in a wider angular range of −90° ≤ θ ≤ 90°. In addition, compared to the patch array, our antenna offers dual-polarization, wider impedance bandwidth (>50%), and higher aperture efficiency. In terms of the electrical size comparison of the two antennas, the patch array has a shorter profile while our antenna has a smaller footprint.

## Figures and Tables

**Table 1:** Performance comparison of OCROP-Flared antennas with different aperture lengths (L_ap_).

L_ap_ (mm)	F (mm)	α	$S{\mathrm{dd}}_{11}\;\leq$ −10 dB				Cross-Pol BW (Peak ≤ −25 dB) (%)	Combined BW (Imp. & Cross-Pol) (%)
			Imp. BW (%)	Gain (dBi)				
				@ 1.5 GHz	Peak	Av.		
165	0	0°	51.8	8.7	10.2	8.8	44.9	40.0
185	41.2	14.0°	51.7	9.4	10.8	9.4	52.1	49.0
205	44.7	26.6°	52.1	9.9	11.2	10.0	59.1	52.1
225	50.0	36.9°	52.4	10.3	11.5	10.3	65.3	52.4
245	56.6	45.0°	52.5	10.7	11.8	10.7	71.9	51.9
265	64.0	51.3°	52.8	10.9	11.7	10.8	36.0	36.0
285	72.1	56.3°	53.1	11.0	11.8	10.9	26.3	26.3

**Table 2:** Performance comparison of OCROP-Flared antennas with different combinations of non-flared (H_sw_) and flared (F_z_) ground cavity sidewall heights.

$L_{ap}$ (mm)	$H_{sw}$ (mm)	$F_{z}$ (mm)	F (mm)	α	S11 ≤ −10 dB				Cross-Pol BW (Peak ≤ −25 dB) (%)	Combined BW (Imp. & Cross-Pol) (%)
					Imp. BW (%)	Gain (dBi)				
						@ 1.5 GHz	Peak	Av.		
265	80	40	64.0	51.3°	52.8	10.9	11.7	10.8	36.0	36.0
	70	50	70.7	45.0°	53.4	11.1	12.2	11.0	105.4	50.3
	60	60	78.1	39.8°	53.8	11.2	12.6	11.2	103.4	47.8
	50	70	86.0	35.5°	53.9	11.4	12.8	11.3	101.3	43.9
	40	80	94.3	32.0°	53.4	11.4	12.9	11.4	66.1	40.6
	30	90	103.0	29.1°	52.6	11.6	13.0	11.4	63.9	38.4

**Table 3:** Dimensions of Antennas #1, #2, and #3 (all dimensions in mm).

Antenna Type	$L_{ap}\;$	$L_{g}$	$F_{xy}$	$H_{cavity}$	$H_{sw}$	$F_{z}$	$w_{r}$
Antenna #1 (α = 0° and F = 0 mm, w/o flare and iris)	165	165	0	120	120	0	N/A
Antenna #2 (α = 35.5° and F = 86.0 mm, w/flare and w/o iris)	265		50		50	70	N/A
Antenna #3 (α = 35.5° and F = 86.0 mm, w/flare and iris)							15, 17.5, 20

**Table 4:** Performance comparison of Antennas #1 (without flare/iris), #2 (with flare, without iris), and #3 (with flare/iris).

Antenna Type	$w_{r}$ (mm)	S_dd11_ ≤ −10 dB					Cross-Pol BW (Peak ≤ −25 dB) (%)	Combined BW (%)	@ 1.89 GHz, $w_{r}=20\;\mathrm{mm}$			
		$f_{1}$~$f_{2}$ (GHz)	Imp. BW (%)	Gain (dBi)					Gain (dBi)	Cross-Pol (dB)	HPBW	
				@ 1.5 GHz	Peak	Av.					E	H
#1	N/A	1.112~1.890	51.0	8.7	10.2	8.8	44.9	40.0	9.9	−32.8	59°	60°
#2	N/A	1.094~1.902	53.9	11.4	12.8	11.3	101.3	43.9	12.8	−18.2	39°	43°
#3	15	1.128~1.911	51.5	11.3	13.4	11.5	77.6	49.0	13.3	−24.2	39°	43°
	17.5	1.136~1.907	50.7	11.3	13.3	11.5	110.4	50.7	13.3	−26.3	39°	43°
	20	1.147~1.905	49.7	11.2	13.3	11.5	82.1	49.7	13.2	−27.7	39°	43°

**Table 5:** OCROP-Flared prototype dimensions (all dimensions in mm).

*L* = *W* = *H*	*L_g_*	*H_sw_*	*g*	*s*	l	*w*	$l^{\prime }$	*w’*	*F_z_*	*L_ap_*	*F*	*α*
51	165	80	11	3	53	30	1	3.6	40	211.2	46.2	30°

**Table 6:** Performance comparison of the proposed antenna with other high-gain antennas.

Antenna Type	Polarization	Aperture Area	Antenna Height	Imp. BW (%)	Peak Gain across imp. BW (dBi)	Peak Cross-Polarization (within −90° ≤ θ ≤ 90°, 0° ≤ φ ≤ 360°) across imp. BW (dB)	Aperture Efficiency @ f_c_ (%)	Prototype Weight (g)
Standard gain horn [22]	Single	3.867$\lambda_{c}{}^{2}$	3.507λ_c_	41.1	16.5	-	57.9	7257.5
Patch array [6]	Single	5.190$\lambda_{c}{}^{2}$	0.161λ_c_	12.9	16.3	≥0 dB	66.0	-
This work	Dual	1.497$\lambda_{c}{}^{2}$	0.609λ_c_	50.7	13.3	≤−25.8	73.4	258.0 g

## Data Availability

Data available within the article.
